# Vaccine Virus

**Published:** 1873-01

**Authors:** 


VACCINE VIRUS.—There has been considerable trouble experienced for some time past to obtain pure and reliable vaccine virus, and physicians will be pleased to learn that Mr. WM. H. PEABODY has made arrangements whereby he will have constantly on hand a stock of pure and reliable virus.

